# Contrast enhanced transesophageal echocardiography in patients with atrial fibrillation referred to electrical cardioversion improves atrial thrombus detection and may reduce associated thromboembolic events

**DOI:** 10.1186/1476-7120-11-1

**Published:** 2013-01-07

**Authors:** Philip H Jung, Marisa Mueller, Christoph Schuhmann, Madeleine Eickhoff, Philip Schneider, Gueler Seemueller, Raphael Dutton, Johannes Rieber, Stefan Kääb, Hae-Young Sohn

**Affiliations:** 1 Medizinische Klinik und Poliklinik I, Klinikum der Universität München, Ziemssenstrasse 1, 80336, Munich, Germany

**Keywords:** Atrial fibrillation, Atrial thrombus, Contrast echocardiography, Transesophageal echocardiography, Cardioversion, Thromboembolic events

## Abstract

**Aims:**

Transesophageal echocardiography (TEE) is the gold standard for the detection of thrombi in patients with atrial fibrillation (AF) before undergoing early electrical cardioversion (CV). However, TEE generates inconclusive results in a considerable number of patients. This study investigated the influence of contrast enhancement on interpretability of TEE for the detection of left atrial (LA) thrombi compared to conventional TEE and assessed, whether there are differences in the rate of thromboembolic events after electrical cardioversion.

**Methods:**

Of 180 patients with AF (51 females, 65.2±13 years) who were referred to CV, 90 were examined with native imaging and contrast enhancement within the same examination (group 1), and 90 were examined with native TEE alone and served as control (group 2). Cineloops of the multiplane examination of the LA and LA appendage (LAA) were stored digitally before and, in group 1, after intravenous bolus application of a transpulmonary contrast agent. Images of group 1 were assessed offline and the diagnosis of LA thrombi was made semi-quantitatively: 1= thrombus present; 2=inconclusive result; 3=no thrombus. The presence of spontaneous echocontrast (SEC) was registered and flow velocity in the LA appendage (LAA-flow) was measured. All patients in whom CV was performed were followed up for 1 year or until relapse of AF. CV related adverse events were defined as any thromboembolic event within 1 week after CV.

**Results:**

No serious adverse events occurred during TEE and contrast enhanced imaging. In group 1 atrial thrombi were diagnosed in 14 (15.6%) during native and in 10 (11.1%) patients during contrast enhanced imaging (p<0.001). Of the 10 patients with thrombi in the contrast TEE group, 7 revealed a decreased LAA-flow (≤0,3m/s) and 8 showed moderate or marked SEC. Uncertain results were significantly more common during native imaging than with contrast enhanced TEE (16 vs. 5 patients, p<0.01). Thrombi could definitely be excluded in 60 (66.7%) during conventional and in 75 patients (83.3%) during contrast enhanced TEE (p<0.01). CV was performed subsequently after exclusion of thrombi and at the discretion of the investigator. In group 1, 74 patients (82.2%) were cardioverted and no patient suffered a CV related complication (p=0.084). In group 2, 76 patients (84.4%) underwent CV, of whom 3 suffered a thromboembolic complication after CV (2 strokes, 1 peripheral embolism).

**Conclusion:**

In patients with AF planned for CV contrast enhancement renders TEE images more interpretable, facilitates the exclusion of atrial thrombi and may reduce the rate of embolic adverse events.

## Background

Transpulmonary contrast agents improve endocardial border delineation of left sided cardiac chambers. Is has been predominantly used for the depiction of the left ventricle and identification of wall motion abnormalities and cardiac masses [1-3]. Despite preliminary promising data for the use of a transpulmonary ultrasound contrast agent for the visualization of the left atrium (LA) and left atrial appendage (LAA) during transesophageal echocardiography (TEE) [4-6], its application for the detection or exclusion of thrombi in patients with atrial fibrillation prior to cardioversion has not been implemented into clinical routine. Several reasons may account for this circumstance. First, clinicians may belief that native TEE alone or with consideration of predictive markers of thrombus formation such as pulsed wave Doppler measurement of the blood flow in the LAA (LAA-flow) and assessment of spontaneous echo contrast (SEC) allows for the save exclusion of atrial thrombi in all patients. Furthermore, the prevalence of a LAA thrombus is low with only 5-13% [7,8] in patients with atrial fibrillation and without therapeutic anticoagulation and the risk of an embolic event after cardioversion is even lower [9,10]. Moreover, the application of an ultrasound contrast agent increases the examination time and expenses.

However, even with the use of TEE and surrogate parameters such as SEC and LAA-flow, there is an embolic rate of about 1% after electrical cardioversion for atrial fibrillation [9]. In addition, thrombi in the LAA can be present in the absence of SEC [8].

The aim of this study was to determine whether the use of contrast enhancement during TEE can improve the ability to identify or exclude LA and LAA thrombi compared to conventional native imaging in patients with atrial fibrillation (AF) before cardioversion. Furthermore, we assessed if there was a difference in the rate of thromboembolic events after cardioversion compared to a control group.

## Methods

### Patients

The study protocol was designed to conform to the principles outlined in the Declaration of Helsinki. Informed consent was obtained from all patients prior to inclusion into the study. Our study was an observational single-centre study designed on an intention-to-cardiovert basis. 180 consecutive patients with AF and indication for CV were randomized by the investigators within the same study period to either receive a TEE with additional contrast enhancement (group 1) or a conventional native TEE (group 2). The baseline characteristics are shown in Table 1. Exclusion criteria were the presence of other more severe clinical conditions such as instable angina, acute coronary syndrome, lack of patient consent as well as contraindications to CV, to TEE or to application of transpulmonary ultrasound contrast agents. Anticoagulation therapy was not required before TEE but was started after TEE if patients were deemed eligible for CV. In the remaining patients, i.e. those not suitable for CV, anticoagulation was started according to CHADS2-Score and as recommended in the ESC-guidelines for the management of patients with AF [11].

**Table 1:** Patient characteristics (* pro-BNP was not measured routinely; abbreviations see text)

	**Contrast-enhanced TEE**	**Control group**	**p value**
n	90	90	
Age	65.9±12	64.5±13	0.474
female, %	32.2	24.4	0.247
BMI, kg/m^2^	27.9±5.0	26.2±4.1	0.111
history of TIA/Stroke, %	4.8	2.7	0.522
COPD ≥ °II, %	4.8	8.2	0.433
diabetes mellitus, %	21.3	13.7	0.244
hypertension, %	58.7	60.3	0.855
CAD, %	27.7	28	0.968
previous.MI, %	4.8	6.8	0.606
hyperlipoproteinemia, %	20.6	20.3	0.958
heart rate 1/min	91.7±26	92.8±24	0.803
antiocoagulation ≥1 day (prior to TEE, %)	51	48	0.764
antiocoagulation ≥3w (prior to TEE, %)	13	11	0.544
hemoglobinn (g/dl)	14.2±1.7	14.3±1.7	0.834
pro-BNP (pg/ml) *	1817±1700*	*	*
creatinine (mg/dl)	1.1±0.49	1.1±0.28	0.728
potassium (mmol/l)	4.2±0.43	4.2±0.43	0.928
C-reactive protein (mg/dl)	1.1±2.1	1.5±2.9	0.377

### Echocardiography

**Figure 1:**
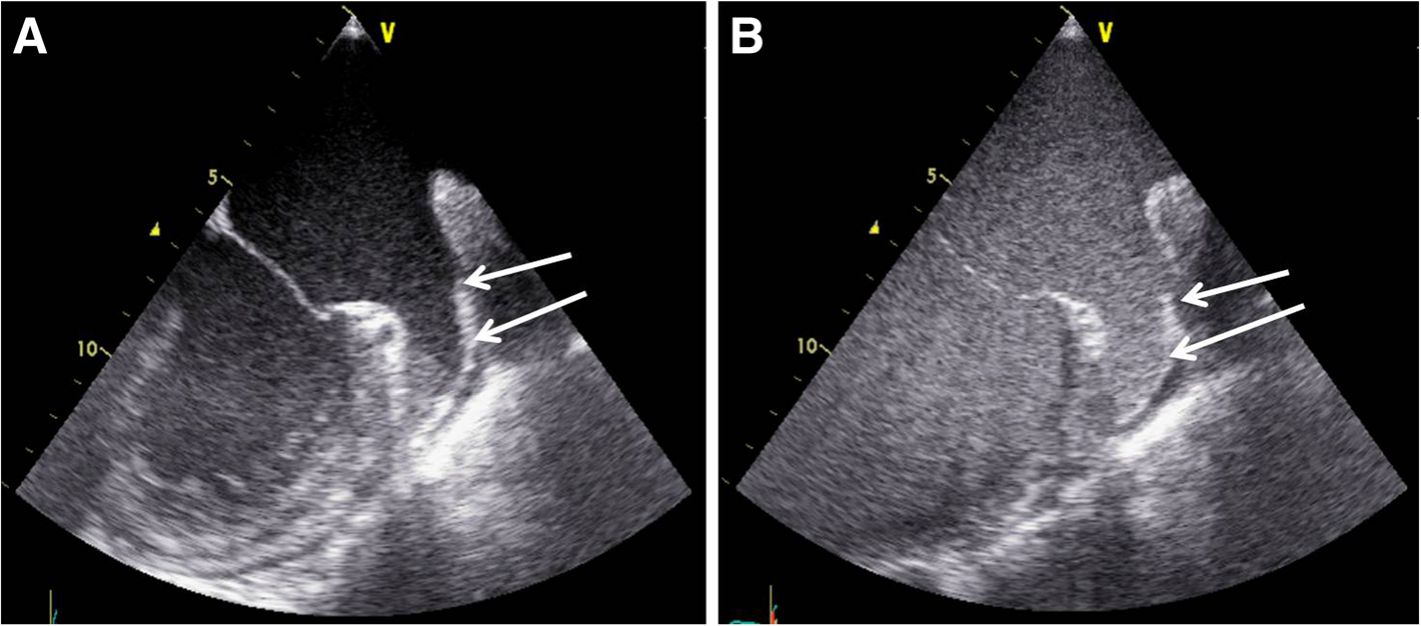
Example of native (A) and contrast enhanced (B) TEE in a patient with atrial fibrillation (arrows: left atrial appendage with sludge-like material in the tip of the LAA during native imaging and homogenous contrast distribution after application of the transpulmonary contrast agent).

Cineloops of the multiplane examination of the LA and LAA were digitally recorded (Philips IE33, GE Vivid VII) throughout each imaging method. Blood flow velocities of the atrial appendage were measured by pulsed wave doppler echocardiography. For the purpose of the present investigation, in group 1 echocardiographic images were acquired before and after bolus application of 1ml contrast agent (SonoVue™ Bracco Diagnostics Inc., Princeton, NJ, USA) into an antecubital vein followed by a bolus of 5-10ml saline according to our standardized study protocol. To avoid shadowing or swirling artefacts, the timing of image acquisition, mechanical index and the setting of the focus zone were individually optimised. Figure 1 depicts examples of native and contrast enhanced TEE.

### Data reading

For the evaluation of interpretability of native versus contrast enhanced TEE, two experienced observers reviewed the digital images during an offline analysis without knowledge of the current patient and the result of the respective other imaging method in group 1. In case of disagreement consensus was sought and achieved in every instance in a second joint session of both observers. The following classification was applied: 1= definite thrombus in at least one imaging plane, 2= inconclusive result in at least one imaging plane and 3= no thrombus in all imaging planes. The images of the patients in group 2 (control group) were only divided into the clinically relevant categories 1=thrombus suspected, CV postponed/refused and 2=no thrombus suspected, CV possible, respectively. Additionally, the prevalence and severity of atrial spontaneous echo contrast was graded semiquantitatively (1=none, 2=mild to moderate and 3=marked).

### Cardioversion and follow-up

Electrical CV was done if no intracavitary thrombus was found or suspected, and postponed in the presence of a thrombus. In less conclusive results, the decision for or against CV was taken at the examiner’s discretion. ECG was monitored during the procedure and for 2 hours after the CV. All patients received anticoagulation treatment starting before and for a minimum of 4 weeks after CV. CV was considered successful if the patient was in sinus rhythm after the procedure for at least 5 beats. In patients with confirmed or suspected thrombus, CV was postponed until anticoagulation had reached an INR-value of ≥2.0 for at least 3 weeks and a subsequent TEE demonstrated disappearance of the thrombus.

All patients were followed up for at least 12 months after CV or until occurrence of a thromboembolic event. The first follow-up was conducted 4 weeks after CV with special anamnestic emphasis on symptoms related to thromboembolic events and recurrence of arrhythmia and clinical cardiac and neurologic examination. In case of suspected thromboembolic event, additional examinations like magnetic resonance tomography were performed. CV related adverse events were defined as any thromboembolic event within 1 week after CV.

### Statistical analysis

The statistical analyses were performed using SPSS statistical software (version 19.0).

The results are given as mean ± standard deviation or percentages where appropriate. Two-by-two tables were constructed to analyse associations between the groups. Significance was tested using Chi-square test for categorical and student t-test for parametric variables. A correction for possible cofounders between the two groups was planned using a propensity adjustment, but no parameter of the baseline characteristics differed significantly (see Table 1). The level of significance for testing all null hypotheses was a two-tailed p-value of 0.05. Since a calculation of the sample size to demonstrate a decrease of 1% of CV related events using contrast enhanced TEE compared to native TEE (80% power, confidence level 95%) estimated a required group of 2500 patients, this was not the primary objective of our study.

## Results

No adverse events related to the application of the contrast agent occurred. No significant differences between both groups in age, comorbidities or baseline laboratory values were found (Table 1). In about half of the patients (group 1: 51%, group 2: 48%) oral anticoagulation or heparin was started at least 1 day prior to TEE, only a minority (group 1: 13%, group 2: 11%) were anticoagulated for at least 3 weeks (Table 1). In all patients anticoagulation was started/continued according to CHADS2-Score and as recommended in the ESC-guidelines for the management of patients with AF [11].

**Figure 2:**
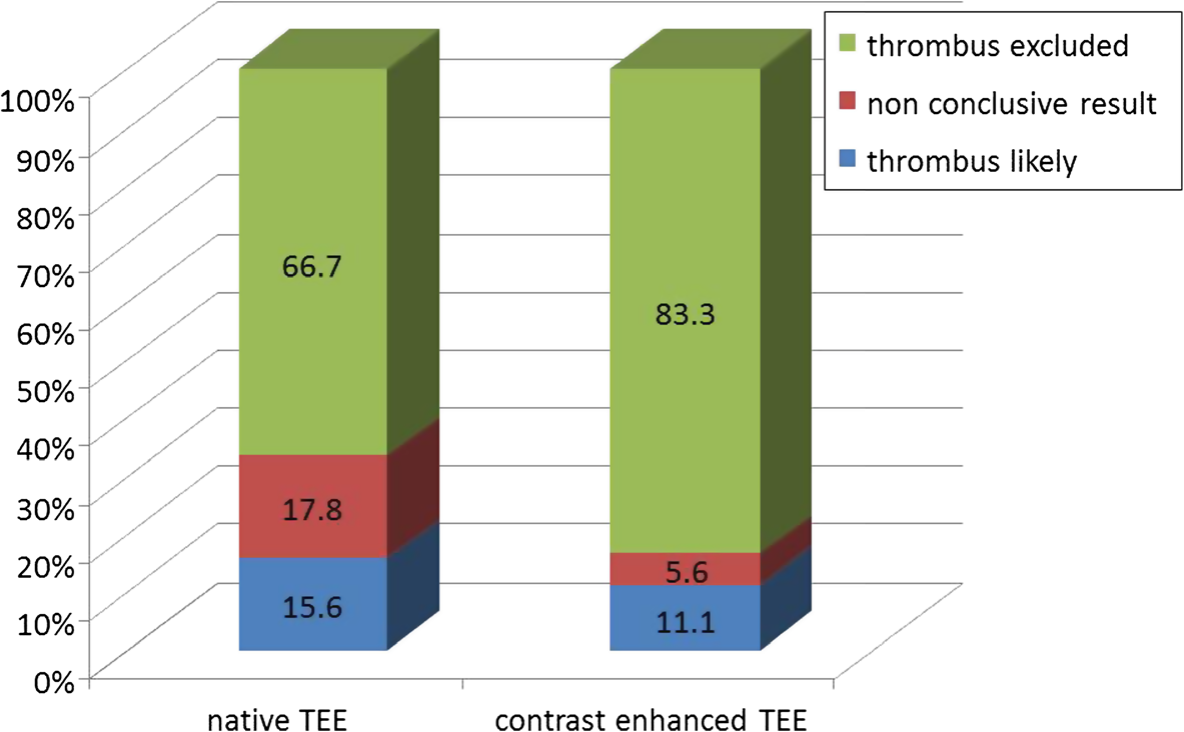
TEE diagnosis before and after application of contrast agent (group 1).

In group 1, the definite existence of a thrombus (classification 1) was assumed in 14 (15.6%) patients during initial examination without contrast enhancement and in 10 (11.1%) patients after the contrast agent was applied (Figure 2). Of the 14 patients with thrombus detection at native imaging, 8 were confirmed by contrast enhanced TEE, 2 remained unclear and in 4 patients a thrombus could be safely excluded and CV performed without subsequent complications. Contrast enhancement allowed for the additional identification of 2 patients with thrombi, in whom native imaging yielded non distinctive findings.

In group 1 inconclusive diagnostic findings, defined as lack of definite identification or exclusion of a thrombus in the multiplane examination of the LA and LAA (classification 2), resulted in 16 patients (17,8%) during native imaging and in 5 (5.6%) patients after application of the contrast agent (p<0.001; Figure 2). In 11 of the 16 patients with previously uncertain results contrast application allowed for exclusion of thrombi, 3 exams remained unclear and in 2 patients a thrombus was newly diagnosed.

Definite exclusion of atrial thrombi using the above mentioned criteria was feasible in 60 (66.7%) during conventional and in 75 patients (83.3%) during contrast enhanced TEE (p<0.001).

The patients in group 2 served as control for subsequent events. Possible results of the TEE were categorized in 1=Thrombus suspected, CV postponed/refused and 2=no thrombus suspected, CV possible, respectively. 10 patients (11.1%) in the control group were supposed to have an atrial thrombus, in the remaining 80 patients (88.9%) no thrombus was suspected of whom 76 (84.4%) were cardioverted subsequently. The success rate of CV was 94.6% in group 1 (70/74 successful CV) and 92.1% in the control group (71/76 successful CV, p=0.73).

We found that in both study groups the average blood flow velocity in the LAA was significantly lower in patients in whom a thrombus could be detected than in those without a thrombus or inconclusive results (group 1: 0.32m/s vs. 0.47m/s, p=0.05; group 2: 0.29m/s vs. 0.48m/s, p=0.03). Accordingly, the presence of moderate or marked spontaneous echo contrast in the LAA was higher in patients with atrial thrombi (group 1: 8 of 10 patients [12] vs. 45 of 80 patients [13], p=0.043; control group: 10 of 10 patients [14] vs. 55 of 80 patients [3], p<0.001).

Of the 10 patients with a definite thrombus after contrast enhanced TEE (group 1), 7 showed a concurrent severe reduction in left atrial appendage blood flow velocity (≤0.3m/s) and in 2 patients no or mild SEC was found.

Cardioversion was carried out at the discretion of the investigator and performed in 74 (82.2%) patients in group 1 and 76 (84.4%) patients in the control group (p=0.514, Table 2). In the control group 3 patients (3.9%) suffered from a CV related thromboembolic event. Of those, 1 patient had an ischemic stroke immediately during cardioversion, 1 patient had an ischemic stroke 2 days after cardioversion and 1 had a peripheral embolism of the A. poplitea 2 days after CV.

**Table 2:** Echocardiographic results and follow up (*1 patient with an event 10 days after CV was excluded due to discontinuation of anticoagulation; abbreviations see text)

	**Contrast-enhanced TEE**	**Control group**	**p value**
n	90	90	
**thrombus detected (%)**	10 (11.1)	10 (11.1)	
LAA-flow (m/s)	0.32 ±0.2	0.29 ±0.2	0.75
LAA-flow ≤ 0.3m/s (%)	7 (70)	8 (80)	0.606
SEC (no/minor-moderate/ marked)	2 / 6 / 2	0 / 4 / 6	0.111
LA diameter (mm)	47.6±7.2	50.1±6.1	0.429
**thrombus excluded / unclear**	80 (88.9)	80 (88.9)	
LAA-flow (m/s)	0.47 ±0.2	0.48 ±0.2	0.711
SEC (no/minor-moderate/ marked)	35 / 38 / 7	25 / 48 / 7	0.243
LA diameter (mm)	46.5±6.3	46.6±7.3	0.895
CV performed (%)	74 (82.2)	76 (84.4)	0.514
CV related events (%)	0 (0)*	3 (3.9)	**0.084**

No CV related adverse event occurred in the contrast enhanced TEE group (p=0.084, Chi-square). However, one patient in the contrast-enhanced TEE-group suffered a peripheral thromboembolic event 10 days after successful CV, but had discontinued anticoagulation against medical advice immediately after CV. This patients TEE had shown marked SEC and depressed LAA blood flow and strict anticoagulation has been advised. Due to study protocol violation (cessation of anticoagulation) and time after CV (embolism beyond predefined time period of CV related events of 1 week), this episode did not account a CV related event.

## Discussion

TEE is the method of choice to exclude thrombus formation in the LA before CV in patients with AF [15], no other imaging modality has so far been able to demonstrate a similar accuracy [16]. Nonetheless, even with the use of TEE and surrogate parameters such as SEC and LAA-flow, there is an embolic rate of about 1% after electrical CV [9] that may be a consequence of suboptimal image quality during TEE. Therefore, it is essential to exclude possible atrial thrombi before the procedure using the best imaging method.

Our patient group was comparable to that of other AF studies regarding age, comorbidities and presence of atrial thrombi [7-9]. In our study, the use of the contrast agent during TEE was feasible and safe, no adverse events related to its application occurred throughout the examination and follow up. This was in good accordance with other studies using the same contrast agent [3,17].

Contrast enhanced TEE permitted the exclusion of thrombi in 4 patients, in whom thrombi were suspected during native imaging. This can be explained by the capability of the contrast agent to completely opacify the LAA even in the presence of artefacts during native imaging leading to falsely identification of thrombi [4]. In 2 patients (2.2%) the contrast application allowed for the additional delineation of atrial thrombi and suspension of the scheduled CV. In these cases, native TEE was not able to detect the atrial thrombi due to insufficient image quality and low degree of echogenicity of the thrombotic material, whereas a persisting contrast-free area in the tip of the LAA during contrast enhanced imaging indicated the presence of a thrombus. In accordance with the results of Recke et al. [4] the number of not conclusive TEE decreased significantly (5.6% vs. 17.8%) using contrast enhancement and therefore facilitated decision making for or against CV.

Some authors suggest that the additional use of surrogate parameters such as the presence of SEC and low LAA-flow allows for the confident identification of patients with low risk of CV related thromboembolic events [18]. However, thrombi in the LAA can be present in the absence of SEC [8]. In our study, 3 of the 10 patients with a definite thrombus after contrast enhanced TEE showed only mild reduction in left atrial appendage blood flow velocity (0.3-0.5m/s) and 2 patients none or only mild SEC. We and other authors therefore conclude that surrogate parameters cannot replace the direct visualisation of the LAA [14] since the duration of AF, the LA size and other factors influence the amount of SEC and LAA-flow.

CV was carried out at the discretion of the investigator and performed in 74 (82.2%) patients in group 1 and 76 (84.4%) patients in the control group (p=0.514, Table 2). A possible explanation for this lack of difference between the groups might be the fact, although not significant (p=0.514), that the contrast application can facilitate the visualisation of the sometimes impressive slow blood flow in the LAA. This effect has also been shown in previous studies [6] and may have led to the decision against CV in some patients in whom atrial function was severely depressed despite exclusion of a thrombus.

The event rate in our study was 3.9% in the control group, where native TEE was performed, and 0% in group 1 using contrast enhancement. Previous studies showed embolic complications of CV in as much as 1% of patients despite exclusion of thrombi with conventional TEE [9]. This corresponds well to the finding of another study comparing results of TEE with intraoperative findings, where 2 of 213 patients had a thrombus despite a normal TEE [19]. In our study the slightly higher event rate in group 2 compared to literature may be a consequence of selection bias or higher cardiac morbidity including patients with valve disease and heart failure. The difference to group 1 (0% event rate) is best explained as a consequence of improved interpretability after contrast application, but did not, however, reach statistical significance (p=0.08). Planned as a single centre study and taking into account a low rate of CV related events, the objective of our study was to test feasibility of contrast enhanced TEE and concomitant improvement of interpretability rather than superiority of contrast enhanced TEE regarding subsequent thromboembolic events.

It can be hypothesised from the above mentioned comparison of TEE and intraoperative findings and another study published by Saeed et al. [10] that even in the presence of an atrial thrombus CV does not necessarily provoke a thromboembolic complication, and that the estimated risk is therefore somewhat lower. This circumstance contributes to the explanation of the low event rate after CV despite the lack of diagnostic accuracy of conventional TEE. The rare appearance of thromboembolism after CV, however, contrasts to its severity with persisting disabling sequelae for the patient. Hence, it appears to be essential to eliminate possible atrial thrombi before the procedure using the best method possible. In our opinion, despite the low event rate, the contrast imaging related increase in duration of the examination and the additional expenses, contrast application should be performed whenever native imaging does not allow for the safe exclusion of atrial thrombi.

## Limitations

The study was conducted on an observational basis without blinding. Therefore, a selection bias as well for the patients as for the examiner may be present. Another possible limitation was that there was no anatomical gold standard to confirm the results of TEE, as could be provided in case of subsequent cardiac surgery and direct visual validation. Furthermore, contrast enhanced TEE cannot accurately differentiate between new and old/organised thrombi, that may not contribute to an increased thromboembolic risk.

The estimated rate for cardioembolic events after cardioversion in the literature is about 1%. On the basis of this low event rate, the estimated sample size to reliably confirm the superiority of contrast enhanced TEE for the prevention of embolic events in patients undergoing CV is far higher than our study group, which was therefore underpowered to show superiority regarding CV related events. Consequently, further examinations should be carried out to clarify the precise benefit of contrast enhancement during TEE in a larger patient group.

## Conclusion

Our study shows that application of an ultrasound contrast agent during TEE is feasible and has the potential of reducing equivocal diagnostic findings for the identification of LA thrombi in patients with AF referred to CV. There was a trend to less thromboembolic complications after CV if a contrast agent was used for the exclusion of thrombi during TEE.

## Competing interests

The authors declare that they have no competing interests.

## Authors’ contributions

PJ initiated the study, recruited patients, carried out most of the echocardiographic studies, contacted patients during follow-up, analysed the data and drafted the manuscript. MM performed echocardiographic examinations and contributed to the writing of the manuscript. CS, ME. PS and GS assisted in the patient recruitment, echocardiographic examinations and performed electrical cardioversions. RD assisted in the echocardiographic examinations and the statistical analysis of the data. JR recruited patients eligible for the study and performed electrical cardioversions. SK conceived of the interpretation of the data and reviewed the manuscript carefully. HYS provided the infrastructure necessary for the realization of the study, conceived of the interpretation of the data and reviewed the manuscript carefully. All authors read and approved the final manuscript.
